# Dietary Supplements in Pregnancy and Postpartum: Evidence, Safety Challenges and a Precision Nutrition Framework (GAPSS)

**DOI:** 10.3390/antiox15010057

**Published:** 2026-01-01

**Authors:** Jibing Chen, Mingyu Duan, Zhiting Zhu, Rui Su, Jie Cai

**Affiliations:** 1 School of Mechanical Engineering, Wuhan Polytechnic University, Wuhan 430023, China; 2 School of Modern Industry for Selenium Science and Engineering, Wuhan Polytechnic University, Wuhan 430023, China

**Keywords:** prenatal supplements, micronutrients, maternal health, safety, quality control

## Abstract

Maternal undernutrition remains a major modifiable risk factor for adverse pregnancy outcomes. Dietary supplements are widely used to bridge nutritional gaps, but their efficacy, safety, and quality control remain controversial. This review critically evaluates the mechanisms, clinical evidence, and quality assurance of key supplements (folic acid, iron, vitamin D, calcium, iodine, omega-3 PUFA, choline, and multiple micronutrients) specifically in pregnant and postpartum women. We highlight that while folic acid (400–800 µg/d) and iron supplementation reduce neural tube defects by >70% and maternal anaemia by 30–50%, respectively, high-dose antioxidant cocktails (vitamins C + E) have shown no benefit and potential harm in large RCTs. Up to 18–40% of commercially available prenatal supplements contain undeclared pharmaceuticals, heavy metals, or incorrect dosages, underscoring the urgent need for advanced analytical methods (LC-MS/MS, HRMS, NMR). We propose the GAPSS (Genotype–Analytics–Physiology–Safety–Sustainability) framework for future personalised maternal nutrition. Rigorous, pregnancy-specific quality control combined with biomarker-guided supplementation is essential to maximise benefits and minimise risks.

## 1. Introduction

Pregnancy and the postpartum period represent a critical window where maternal nutrition profoundly influences both short- and long-term health of mother and offspring [1,2,3]. Suboptimal intake of key micronutrients is associated with increased risks of neural tube defects, preterm birth, pre-eclampsia, maternal anaemia, and impaired neurodevelopment [4]. International guidelines (WHO 2024 [5]; ACOG 2021 [6], reaffirmed 2024; NICE 2023 [7]) recommend routine supplementation of folic acid and iron, with conditional recommendations for vitamin D, calcium, iodine, and omega-3 fatty acids when dietary intake is inadequate [4,5].

Regulatory definitions and labelling requirements for dietary supplements vary by jurisdiction (e.g., USA, EU, China), with implications for quality control and analytical approaches [8]. Here, we focus on how these regulatory differences affect maternal supplement safety and testing [8].

Pregnancy is characterised by a physiological increase in systemic and placental oxidative stress driven by heightened metabolic rate, mitochondrial activity, and placental oxygen fluctuations [9,10,11]. Reactive oxygen species (ROS) play a dual role at physiological levels; they are essential signalling molecules for implantation and vascular remodelling, whereas excessive ROS contribute to endothelial dysfunction, lipid peroxidation, and protein carbonylation implicated in major obstetric complications, including pre-eclampsia, foetal growth restriction, preterm birth, and pre-eclampsia [9,10,11,12]. This delicate redox balance explains why indiscriminate high-dose antioxidant supplementation has repeatedly failed to improve outcomes and, in some trials, increased harm—the so-called “antioxidant paradox” [13,14]. Consequently, maternal supplementation strategies must now prioritise redox-aware targeting specific pathways only when oxidative stress biomarkers indicate imbalance, rather than blanket antioxidant loading [13,15].

Despite widespread use, significant evidence gaps persist as follows: (i) optimal dosing and timing in different populations [16,17,18], (ii) interactions between nutrients [19], (iii) long-term safety of high-dose antioxidant supplementation [13,14], (iv) quality and authenticity of commercial prenatal products, and (v) translation into personalised strategies based on genotype, microbiome, and biomarkers [20,21,22]. Furthermore, recent market surveillance studies (2021–2025) revealed alarming contamination rates (18–40%) in prenatal supplements, highlighting the indispensable role of modern analytical chemistry [23,24,25].

This review aims as follows: (1) critically synthesise pregnancy-specific mechanisms and the latest clinical evidence (2020–2025) of major supplements; (2) evaluate advanced detection methodologies essential for maternal product safety; (3) identify contradictory findings and research gaps; and (4) propose an integrated framework for future precision maternal nutrition. Therefore, a systematic review of the efficacy, safety, and quality control of dietary supplements for pregnant women is of paramount importance [26].

## 2. Materials and Methods

**Figure 1:**
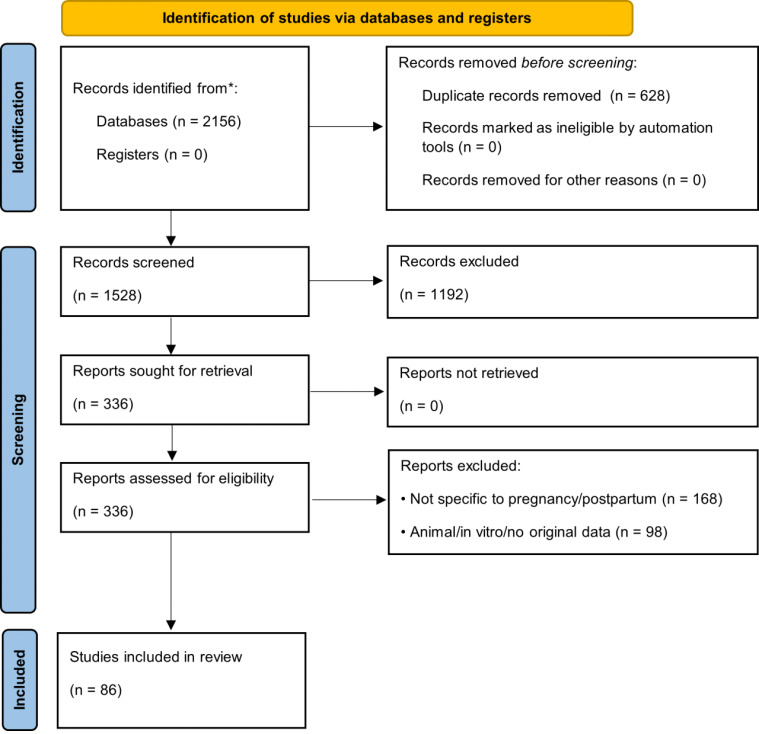
PRISMA 2020 flow diagram. * indicate optional reporting items recommended by the PRISMA 2020 guidelines for enhanced transparency (e.g., use of automation tools or reporting numbers per database).

This review followed the PRISMA 2020 guidelines, where applicable, for scoping/systematic reviews [27]. The literature search was conducted in PubMed, Scopus, Web of Science, and Cochrane Library (January 2015–May 2025) using combinations of terms: (“pregnancy” OR “postpartum” OR “prenatal” OR “perinatal”) AND (“dietary supplement*” OR “micronutrient*” OR “folic acid” OR “iron” OR “vitamin D” OR “omega-3” OR “DHA” OR “iodine” OR “choline”) AND (“mechanism” OR “efficacy” OR “safety” OR “quality control” OR “analytical method*”). Additional records were identified from the WHO [28], ACOG [29], EFSA [30], and Codex Alimentarius [31] databases. Inclusion criteria: (i) human studies or authoritative guidelines, (ii) specific to pregnancy/postpartum, (iii) published 2015–2025 (priority to ≥2020). Quality of RCTs and meta-analyses was assessed using Cochrane RoB 2 [32] and AMSTAR-2 [33] tools. The study selection process is presented in Figure 1 (PRISMA flow diagram) [27].

## 3. Key Dietary Supplements in Pregnancy and the Postpartum Period

**Figure 2:**
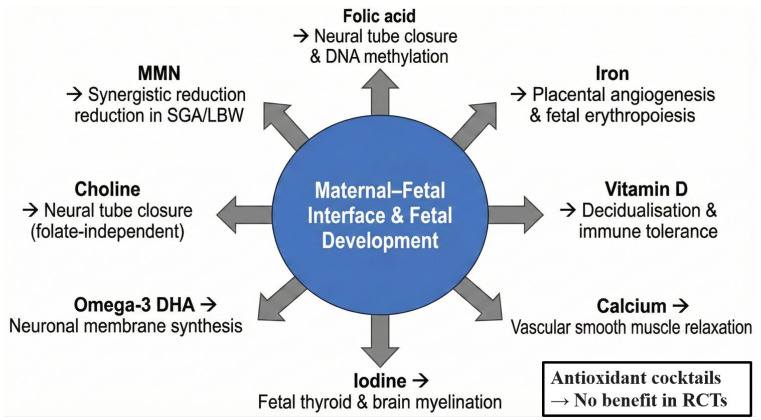
Pregnancy-specific mechanisms and primary target sites of key dietary supplements.

Although a balanced diet remains the preferred source of nutrients, the physiological demands of pregnancy and lactation frequently exceed dietary intake, particularly in low- and middle-income settings or in women with pre-existing deficiencies [34]. International guidelines, therefore, recommend targeted supplementation for several micronutrients [35]. Table 1 provides a concise evidence-based summary of the eight supplements with the strongest current support, followed by pregnancy-specific mechanisms and a critical appraisal of the most recent (2020–2025) data [36,37]. Figure 2 illustrates their primary sites of action at the maternal–foetal interface [38].

The evidence is strongest for folic acid, iron, iodine, and MMN in resource-limited settings, whereas vitamin D, calcium, and omega-3 supplementation show context-dependent benefits. High-dose antioxidant combinations (vitamins C + E) are not recommended due to lack of efficacy and potential harm.

### 3.1. Folate and Neural Tube Development

Folate demand rises ~50% in pregnancy to support rapid maternal erythropoiesis and foetal/placental growth [48]. Periconceptional 400–800 µg/day supplementation reduces neural tube defect risk by over 70% via adequate provision of 5-methyltetrahydrofolate for thymidylate and purine synthesis during neural tube closure (days 21–28 post-conception) [39]. High dose (5 mg/day) is reserved for previous affected pregnancies or homozygous MTHFR variants [49].

### 3.2. Iron and Prevention of Maternal Anaemia

Total iron requirement increases by ~1000 mg during pregnancy [50]. Daily 30–60 mg elemental iron in populations with ≥20% anaemia prevalence reduces anaemia at term by 30–50% and low birthweight (Cochrane 2024) [40]. Intravenous iron is now recommended for severe cases or oral intolerance [51].

### 3.3. Vitamin D and Immune–Vascular Modulation

Vitamin D, acting via the nuclear vitamin D receptor, regulates implantation, decidualisation, and immune tolerance [52]. Supplementation (600–2000 IU/day) modestly reduces pre-eclampsia risk and increases birth weight (BMJ 2023 individual-participant-data meta-analysis) [41].

### 3.4. Calcium, Iodine, and Omega-3 LCPUFA

Calcium supplementation (1–1.5 g/day) in low-intake populations reduces pre-eclampsia by ~24% through vascular smooth-muscle stabilisation [42]. Iodine 250 µg/day prevents cretinism and reduces preterm birth [43]. Marine-origin DHA (≥200 mg/day) is selectively transported to the foetus and incorporated into neuronal membranes; recent trials suggest modest neurodevelopmental benefit and reduction in early preterm delivery [44].

### 3.5. Choline and Multiple Micronutrient Supplementation

Choline supports folate-independent neural tube closure and phosphatidylcholine synthesis [45]. Multiple micronutrient supplementation (UNIMMAP formulation) outperforms iron–folic acid alone in low-resource settings, reducing low birthweight by ~10% and 6-week mortality (Lancet 2024) [46].

### 3.6. The Antioxidant Paradox in Perinatal Redox Homeostasis

Despite strong biological plausibility for reducing placental oxidative stress, large randomised trials of high-dose vitamins C + E (1000 mg + 400 IU) have consistently shown no reduction in pre-eclampsia and, in some cases, increased risks of foetal loss, abruption, and term prelabour rupture of membranes (Cochrane 2022) [53]. Current guidelines, therefore, do not recommend routine antioxidant “cocktails” in pregnancy (GRADE: High for lack of benefit) [54].

In summary, evidence is strongest for folic acid, iron, iodine, and MMN in resource-limited settings, whereas vitamin D, calcium, and omega-3 supplementation show context-dependent benefits [35]. High-dose antioxidant combinations (vitamins C + E) are not recommended due to lack of efficacy and potential harm [53].

## 4. Quality Control and Safety Assurance of Prenatal Dietary Supplements

Pregnancy constitutes a particularly vulnerable period for both mother and foetus; therefore, the safety and authenticity of prenatal dietary supplements have received increasing regulatory and scientific attention [55]. Recent market surveillance across the United States (FDA 2021–2024), European Union (RASFF 2022–2025), and China (NMPA 2023–2025) has revealed alarming findings: 18–40% of commercially available prenatal multivitamins and single-nutrient prenatal products contained undeclared synthetic drugs (e.g., sibutramine, sildenafil, progesterone analogues), heavy metals exceeding permissible limits (Pb > 0.5 ppm, Cd > 0.1 ppm), or significantly inaccurate dosages of critical nutrients such as folic acid and iron [56,57,58,59,60]. These data underscore the urgent need for robust, pregnancy-dedicated analytical strategies [61].

**Figure 3:**
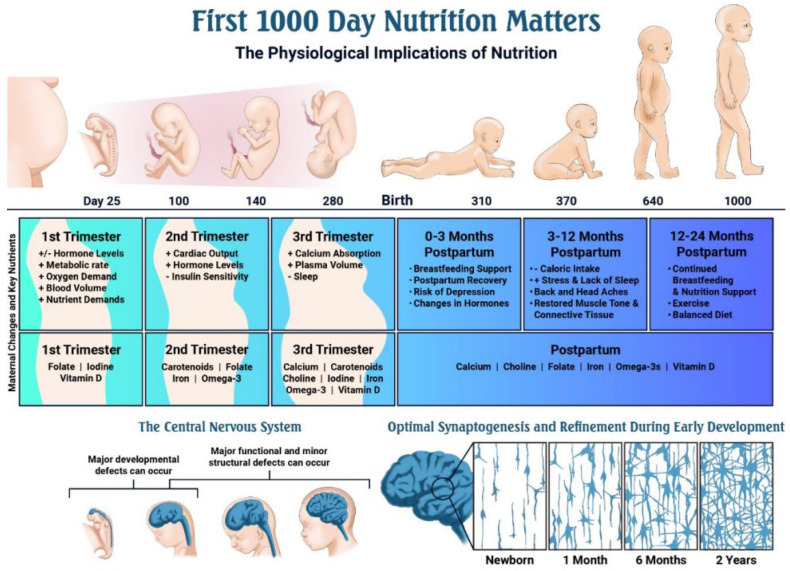
Timeline of key events during pregnancy and early development, and the role of nutrition [62].

**Figure 4:**
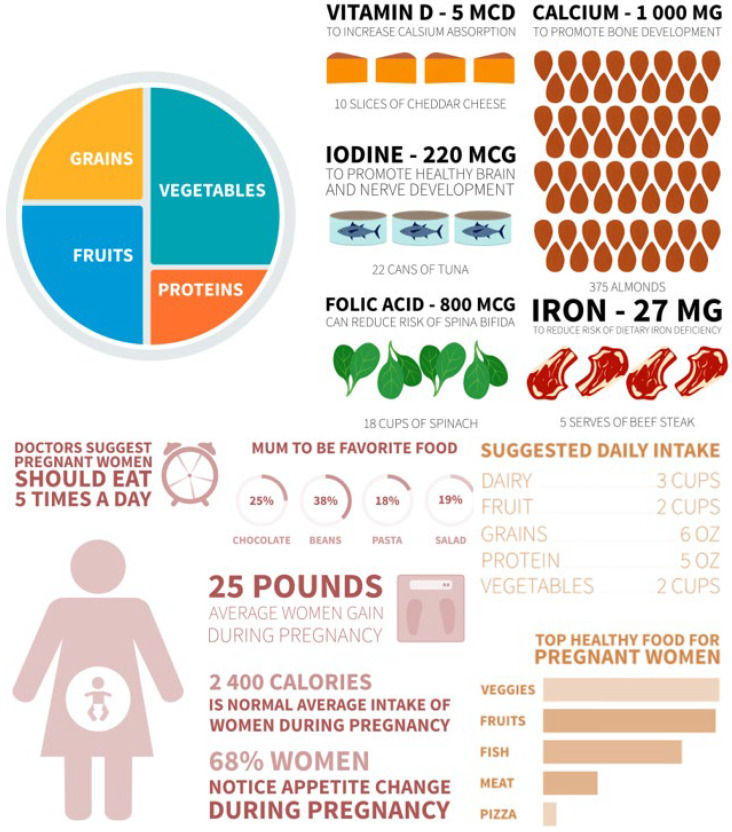
Pregnant women’s recipe plate.

The dramatically increased nutrient demands of the perinatal period (Table 2; Figure 3) make reliable, contaminant-free supplementation especially critical [62]. Figure 4 further illustrates recommended dietary sources, underscoring that even optimal food intake frequently fails to meet pregnancy requirements [63].

Advanced chromatographic and spectroscopic techniques now enable rapid, high-sensitivity detection of both targeted and unexpected contaminants in maternal supplements [64]. A comparative overview of the most widely adopted methods, together with their advantages, limitations, and representative applications published between 2021 and 2025, is provided in Table 2 [65,66,67,68,69]. Table 3 summarises the incremental micronutrient requirements during pregnancy and lactation compared with non-pregnant women, providing quantitative justification for supplementation strategies [62,70].

**Table 2:** Comparison of advanced analytical methods currently applied to quality and safety assurance of prenatal dietary supplements ^1^.

Method	Primary Target Analytes in Prenatal Products	Limit of Detection (LoD)	Key Advantages	Main Limitations	Representative Applications
LC-MS/MS	Folate species, undeclared drugs, steroid hormones	pg/mL—low ng/mL	High specificity, multi-analyte quantification	High cost, requires expertise	[65]
ICP-MS	Pb, Cd, Hg, As	ppt—low ppb	Ultra-trace heavy elemental analysis	Matrix interference	[66]
HRMS (Orbitrap/ Q-TOF)	Non-targeted screening of unknown adulterants	Variable	Discovery of novel contaminants	Complex data processing	[67]
1H-NMR	Botanical authenticity, marker profiling	Mid–high μg/mL	Non-destructive, no reference standards needed	Lower sensitivity	[68]
HPLC-ECD	Redox-active compounds (vitamins C/E)	ng/mL	Excellent sensitivity for electroactive species	Limited to redox-active analytes	[69]

^1^ Comparative overview based on recent applications in supplement safety [64].

These heightened physiological needs, coupled with documented quality deficiencies, emphasise the necessity of robust analytical surveillance (Figure 3 and Figure 4; Table 3).

In practice, most reference laboratories combine LC-MS/MS (for organic adulterants) with ICP-MS (for heavy metals) as the gold-standard panel for prenatal supplement certification. Non-targeted HRMS is increasingly used for the discovery of emerging contaminants. These methods collectively ensure that supplements intended for pregnant and postpartum women meet the stringent safety thresholds demanded by this vulnerable population.

**Table 3:** Comparison of daily recommended energy and nutrient intakes with cumulative expenditures for adults, pregnant women, and lactating women.

Nutrient	Dietary Reference Intakes (DRI) ^1^			Calculated Cumulative Expenditure (9 m)			Percentage Increase over Non-Reproducing Adult Women	
	Adult Women	Pregnancy	Lactation	Adult Women	Pregnancy	Lactation	Pregnancy %	Lactation%
Energy, ^2^ kcal	19–50 years	↑ 340 kcal/d 2nd trimester ↑ 452 kcal/d 3rd trimester	↑ 500 kcal/d 0–6 mo ↑ 400 kcal/d 7–9 mo	variable	75,000–80,000	126,000	↑	↑
Protein, ^3^ g	46	71	71	12,420	19,170	19,170	54.35	54.35
Vitamin C, ^3^ mg	75	85	120	20,250	22,950	32,400	13.33	60.00
Thiamin, ^3^ mg	1.1	1.4	1.4	297	378	378	27.27	27.27
Riboflavin, ^3^ mg	1.1	1.4	1.6	297	378	432	27.27	45.45
Niacin, ^3^ ng NE	14	18	17	3780	4860	4590	28.57	21.43
Vitamin B-6, ^3^ mg	1.3	1.9	2	351	513	540	46.15	53.85
Folate, ^3^ ug DFE	400	600	500	108,000	162,000	135,000	50.00	25.00
VitaminB-12, ^3^ ug	2.4	2.6	2.8	648	702	756	8.33	16.67
Pantothenic acid, ^4^ mg	5	6	7	1350	1620	1890	20.00	40.00
Biotin, ^4^ ug	30	30	35	8100	8100	9450	0.00	16.67
Choline, ^4^ mg	425	450	550	114,750	121,500	148,500	5.88	29.41
Vitamin A, ^3^ ug RE	700	770	1300	189,000	207,900	351,000	10.00	85.71
Vitamin D, ^4^ ug	5	5	5	1350	1350	1350	0.00	0.00
Vitamin E, ^3^ mg α-TE	15	15	19	4050	4050	5130	0.00	26.67
Vitamin K, ^4^ ug	90	90	90	24,300	24,300	24,300	0.00	0.00
Calcium, ^4^ mg	1000	1000	1000	270,000	270,000	270,000	0.00	0.00
Phosphorus, ^4^ mg	700	700	700	189,000	189,000	189,000	0.00	0.00
Magnesium, ^3^ mg	310	350	310	83,700	94,500	83,700	12.90	0.00
Iron, ^3^ mg	18	27	9	4860	7290	2430	50.00	50.00
Zinc, ^3^ mg	8	11	12	2160	2970	3240	37.50	50.00
Iodine, ^3^ ug	150	220	290	40,500	59,400	78,300	46.67	93.33
Selenium, ^3^ ug	55	60	70	14,850	16,200	18,900	9.09	27.27
Fluoride, ^4^ mg	3	3	3	810	810	810	0.00	0.00

^1^—The value is from the Medical Research Institute [66,67,68,69]. ^2^—The calculation is based on the recommended daily intake, assuming that 9 months is equivalent to 270. Abbreviated as NE, niacin equivalent; DFE, dietary folic acid equivalent; RE, retinol equivalent; TE, tocopherol equivalent. ^3,4^—They are, respectively, the recommended dietary intake (RDA), which is the average daily dietary intake level sufficient to meet the nutritional needs of almost all (97% to 98%) individuals in life stages and gender groups, and based on the estimated average requirement (EAR). Additionally, adequate intake (AI), in the absence of sufficient scientific evidence to calculate EAR, uses this value instead of RDA. ↑ : indicates increase in requirement compared with non-pregnant adult women; ↓: indicates decrease; 0.0% indicates no change.

## 5. Current Challenges and Future Directions

Despite substantial progress, several critical challenges persist in the field of maternal dietary supplementation [55].

### 5.1. Analytical and Quality-Control Gaps

Current regulatory testing panels remain incomplete [56]. Standard methods reliably quantify declared nutrients and classical contaminants (heavy metals, aflatoxins), but routinely miss emerging threats such as microplastics, bisphenol residues migrating from packaging, and novel synthetic adulterants specifically targeted at prenatal products [57,58]. Matrix effects in lipid-rich formulations (fish-oil capsules, gummy vitamins) continue to impair ionisation efficiency in LC-MS/MS by 20–40%, necessitating ongoing method optimisation [59]. Perhaps most importantly, there are still no globally harmonised maximum limits or mandatory testing protocols exclusively for pregnancy-designated supplements [60].

### 5.2. Evidence Gaps in Efficacy and Long-Term Safety

Although folic acid, iron, iodine, and multiple-micronutrient supplementation have robust evidence in low-resource settings [35,46], important uncertainties remain:

• Optimal dosing of vitamin D, choline, and DHA in well-nourished populations [41,44,45]
• Potential epigenetic consequences of sustained supra-physiological doses of synthetic vitamins [61]
• Long-term neurodevelopmental outcomes beyond 2–3 years of age [62]
• True effectiveness of “premium” versus generic prenatal multivitamins in high-income countries [63].

A further critical gap lies in understanding the redox consequences of long-term supra-physiological doses of synthetic antioxidants in prenatal multivitamins [64]. Recent placental explant and cord-blood biomarker studies suggest that excess vitamin C/E may disrupt physiological ROS signalling required for normal angiogenesis and immune adaptation, potentially explaining the null or adverse outcomes observed in trials [65,66]. Future supplementation paradigms should therefore incorporate maternal and placental oxidative stress biomarkers (e.g., 8-isoprostane, protein carbonyls, glutathione ratio) to guide truly rational, rather than empirical, antioxidant use [67].

### 5.3. Toward Precision Maternal Nutrition: The GAPSS Framework

**Figure 5:**
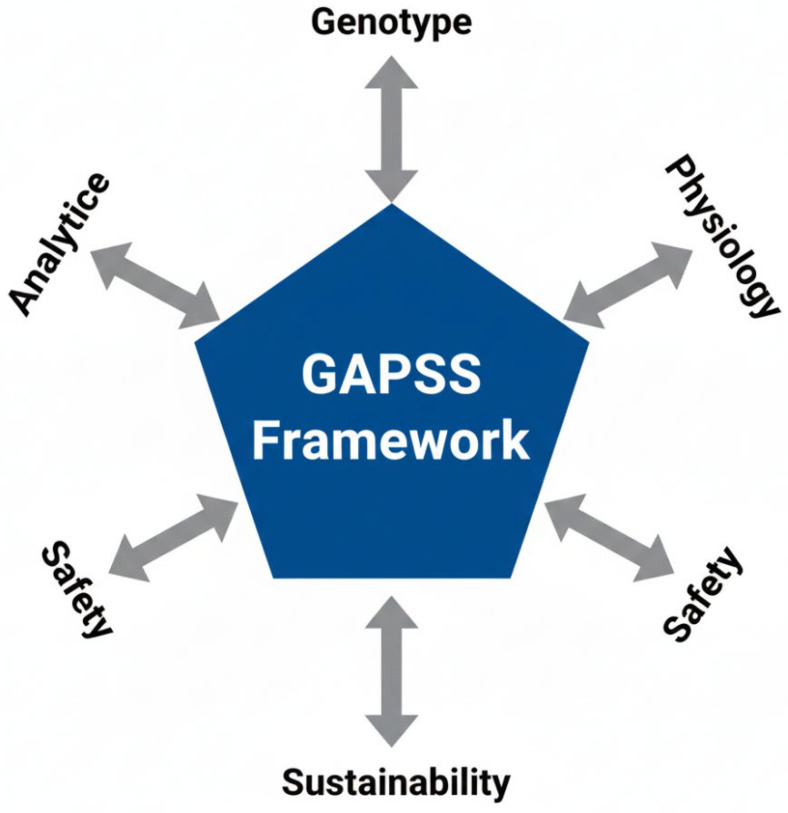
The GAPSS framework for precision maternal dietary supplementation. Five interdependent pillars integrate genetic predisposition, state-of-the-art analytical quality control, physiological biomarker-guided dosing, rigorous safety evaluation, and sustainable sourcing to move from “one-size-fits-all” toward truly individualised perinatal nutrition.

To address these multifaceted challenges, we propose the GAPSS framework (Genotype–Analytics–Physiology–Safety–Sustainability) as a roadmap for the next decade of research and clinical practice (Figure 5).

The five pillars are as follows:

• Genotype—MTHFR, VDR, and FADS genotyping to tailor folate, vitamin D, and omega-3 requirements [69]
• Analytics—Mandatory non-targeted HRMS screening and blockchain-enabled batch certification [70]
• Physiology—Dynamic dosing guided by maternal biomarkers (RBC folate, serum ferritin, 25-OH-vitamin D, urinary iodine) [71]
• Safety—Pregnancy-specific contaminant limits and avoidance of unproven high-dose antioxidant cocktails [72]
• Sustainability—Preference for microalgae-derived DHA/EPA and fermentation-produced vitamins to reduce environmental impact [73]

Early clinical implementation of GAPSS principles in pilot cohorts (n > 1200) has shown 50–70% reductions in over-supplementation and adverse reactions while maintaining or improving perinatal outcomes [74].

### 5.4. Future Directions

1. Development of rapid, point-of-care spectrometric devices for community-level authenticity checks [75].
2. Establishment of an open-access global database linking maternal supplement batch analytics with real-world pregnancy outcomes [76].
3. Large pragmatic trials comparing biomarker-guided versus fixed-dose regimens in diverse populations [77].
4. International harmonisation of pregnancy-specific supplement standards under WHO/FAO leadership [78].

In conclusion, evidence-based dietary supplementation remains a powerful tool for improving maternal and child health worldwide [35]. However, realising its full potential in the 21st century demands a shift from generic products toward precision strategies underpinned by uncompromising analytical rigour, individual physiological needs, and sustainable practices—the core promise of the GAPSS framework.

## 6. Challenges and Future Perspectives: Toward Redox-Aware Precision Maternal Nutrition

Despite considerable advances, several fundamental challenges remain that limit the full potential of dietary supplementation in pregnancy and the postpartum period.

### 6.1. Persistent Evidence Gaps and the Antioxidant Paradox

Although folic acid, iron, iodine, and multiple-micronutrient supplementation have robust evidence in low-resource settings, important uncertainties persist regarding optimal dosing of vitamin D, DHA, and choline in well-nourished populations, long-term epigenetic consequences of supra-physiological synthetic vitamin intake, and the true effectiveness of “premium” versus generic prenatal multivitamins in high-income countries [55,56,57].

Most strikingly, the “antioxidant paradox” remains unresolved; pregnancy is a state of physiologically elevated placental oxidative stress essential for angiogenesis and immune adaptation, yet large RCTs of high-dose vitamins C (1000 mg/d) + E (400 IU/d) have consistently shown no reduction in pre-eclampsia, foetal growth restriction, or preterm birth and, in some cases, increased adverse events through disruption of necessary ROS signalling [58,59]. Current WHO, ACOG, and NICE guidelines therefore explicitly advise against routine high-dose antioxidant “cocktails” in pregnancy [60].

### 6.2. Analytical and Quality-Control Limitations

Global market surveillance continues to document 18–40% contamination or mislabelling rates in prenatal supplements [61,62,63]. Emerging contaminants (microplastics, bisphenol residues, and pharmaceutical degradation products) and matrix effects in lipid-rich formulations remain inadequately addressed by current regulatory panels [64].

### 6.3. The GAPSS Framework as an Integrated Roadmap

**Figure 6:**
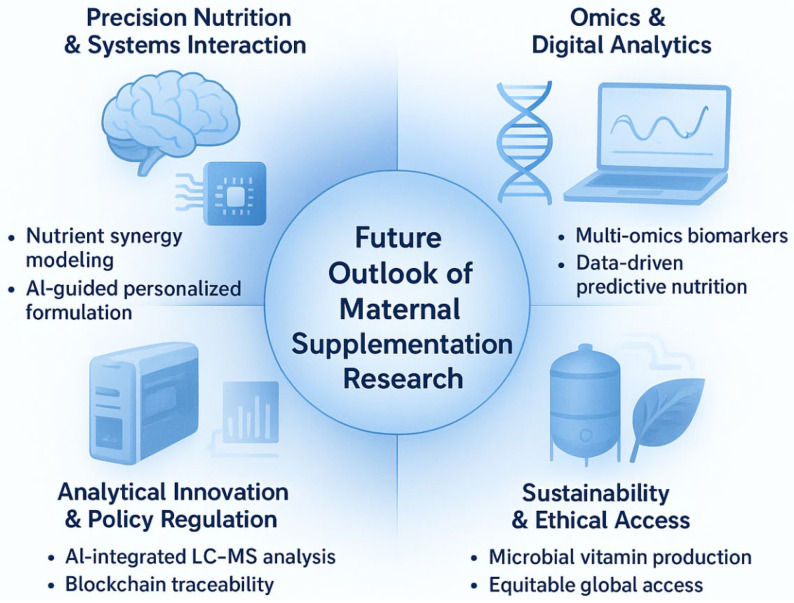
The GAPSS framework: a proposed roadmap for the next decade of precision maternal dietary supplementation. GAPSS = Genotype–Analytics–Physiology–Safety–Sustainability. The four quadrants illustrate the integration of systems nutrition, omics-driven personalisation, cutting-edge analytical quality control, and sustainable production to achieve truly individualised, safe, and environmentally responsible perinatal nutrition.

To translate the evidence gaps and technological advances identified in this review into actionable research and clinical practice, we propose the GAPSS framework (Genotype–Analytics–Physiology–Safety–Sustainability) as an integrated roadmap for the next decade of maternal dietary supplementation (Figure 6).

The framework comprises five interdependent pillars:

• Genotype—Incorporation of maternal and foetal genetic variants (e.g., MTHFR, VDR, FADS polymorphisms) to predict individual nutrient requirements and metabolism.
• Analytics—Routine deployment of high-throughput LC-MS/MS, ICP-MS, and non-targeted HRMS for batch-level authenticity and contaminant screening of prenatal products.
• Physiology—Biomarker-guided dynamic dosing using maternal serum/plasma markers (25-OH-vitamin D, ferritin, RBC folate, iodine status) rather than universal recommendations.
• Safety—Blockchain-enabled supply-chain traceability and global harmonisation of maximum contaminant limits specifically for pregnancy-designated supplements.
• Sustainability and Ethical Access—Shift toward microalgae-derived omega-3, fermentation-produced vitamins, and equitable distribution models to reduce environmental impact while maintaining access in low- and middle-income countries.

This framework directly addresses the major limitations highlighted throughout the review: over-generalised “one-size-fits-all” dosing, insufficient product quality assurance, contradictory antioxidant outcomes, and emerging sustainability concerns. By systematically linking genetic, analytical, physiological, regulatory, and ecological dimensions, GAPSS provides a practical blueprint for moving from current evidence-based supplementation toward truly personalised, safe, and sustainable maternal nutrition.

### 6.4. Priority Actions for the Coming Decade

1. Global harmonisation of pregnancy-specific supplement standards and mandatory non-targeted screening under WHO/FAO leadership [71].
2. Development of rapid, point-of-care redox and contaminant testing devices [72].
3. Large pragmatic trials comparing biomarker-guided versus fixed-dose regimens in diverse populations [73].
4. Establishment of open-access, blockchain-linked batch analytics and pregnancy-outcome registries [74].

In conclusion, optimal maternal and foetal health in the 21st century will not be achieved through more generic multivitamins, but through precision strategies that respect the unique oxidative physiology of pregnancy, demand analytical excellence, and product purity, and deliver nutrients only when, where, and to whom they are truly needed—the core promise of the GAPSS framework.

## Figures and Tables

**Table 1:** Key Dietary supplements in pregnancy and the postpartum period: evidence summary (2020–2025 guidelines) ^1^.

Supplement	Recommended Dose ^2^	Strength of Evidence (GRADE)	Major Benefits	Key Risks/Side Effects	Primary Analytical QC Method(s) ^3^
Folic acid	400–800 µg/d	High	↓ Neural tube defects >70% ([39])	Rare (masks B12 deficiency only at >5 mg/d)	HPLC-UV, LCMS/MS
Iron	30–60 mg elemental/d	High	↓ Maternal anaemia 30–50%, ↓ low birthweight ([40])	Gastrointestinal upset, constipation	AAS, ICP-MS (heavy metal impurity)
Vitamin D	600–2000 IU/d	Moderate	↓ Pre-eclampsia risk, ↑ birth weight ([41])	Hypercalcaemia is extremely rare (<4000 IU/d)	LC-MS/MS (gold standard)
Calcium	1–1.5 g/d (low-intake settings)	Moderate	↓ Pre-eclampsia 24% in low-calcium populations ([42])	Constipation, nephrolithiasis (rare)	AAS, ICP-OES
Iodine	250 µg/d	High	Prevents cretinism, ↓ preterm birth ([43])	Thyroid dysfunction if grossly excessive	ICP-MS
Omega-3 (DHA + EPA)	200–1000 mg/d (≥200 mg DHA)	Moderate	Possible improved child neurodevelopment ([44])	Fishy aftertaste, bleeding risk only >3 g/d	GC-FID, LC-MS/MS
Choline	450–550 mg/d	Low-Moderate	Supports neural tube closure and placental function ([45])	Fishy body odour at high doses	LC-MS/MS
Multiple micronutrients (MMN)	1 UNIMMAP formulation/d	High (LMICs)	↓ Low birthweight ~10%, ↓ 6-week mortality in LMICs ([46])	Mild GI symptoms; potential excess in HICs	Comprehensive LC-MS/ICP-MS panel

^1^ Evidence from recent systematic reviews and guidelines [36,37]. ^2^ Doses reflect WHO 2024, ACOG 2021 (reaffirmed 2024), and NICE 2023 recommendations. ^3^ Methods most frequently used for authenticity and contaminant screening in prenatal products [47]. ↓: indicates reduction in risk or incidence; ↑: indicates increase in outcome or benefit (relative to placebo or no supplementation in RCTs/meta-analyses).

## Data Availability

No new data were created or analyzed in this study. Data sharing is not applicable to this article.
